# Supplementary material for: D-CyPre: a machine learning-based tool for accurate prediction of human CYP450 enzyme metabolic sites
Source: PeerJ Comput Sci. 2024 May 7;10:e2040. doi: 10.7717/peerj-cs.2040 (PMC11157575; doi:10.7717/peerj-cs.2040)

Supporting Information

For several steps used in the training process, such as using nine or one subtypes of data to train the model of one subtype, some attempts and comparisons were also made in this study. First, we compared the performance of feature generators trained on data from a single enzyme versus nine enzymes (Table S11). The results indicated that the model trained on nine enzymes had stronger generalization capability. To examine differences in atom and bond feature distributions in chemical space when training a single discriminator with atom and bond, we used t-SNE to visualize these features (Figure S1). Here, we focus our discussion on the differences of 2B6 and 2C9, which represent two distinct distributions. The results show that when training the 2B6 model, the distribution of atoms and bonds is almost as distinct as when using atom and bond data separately. However, when building the 2C9 model, we observed a cross-distribution between the two types of data. This indicates that some atoms and bonds share similar information that is not present in other atoms. This may allow the model to learn more diverse knowledge during training. This suggests that our model performs at least as well as models based solely on atoms or bonds. However, models built using only atom or bond data have the unique advantage of being able to optimize parameters separately, potentially resulting in higher Jaccard Scores. We compared the performance of these two approaches (Table S12) and found that using atom and bond data separately had a slight advantage, which has the potential to augment further as the dataset grows in magnitude. We also examined the performance of our models on data containing non-reactants (Table S13) and found a significant decrease in Jaccard Score and Precision. We believe this is due to the fact that our training data only included reactants and did not account for the vast chemical space of non-reactants. Introducing a large number of non-reactants may negatively impact model performance when reactant data is limited. This issue may require increasing the size of our database to resolve.

Since a new XGBOOST model must be built in every epoch to output the Jaccard Score of D-CyPre, the training process for the entire model becomes extremely slow. This is not conducive to finding parameters with high Jaccard Score. If only D-MPNN with high Jaccard Score calculated by the output of a single-layer neural network is used to build XGBOOST, the training time for the model will be significantly reduced. To evaluate whether there is a difference in training results between these two methods, we recorded the Jaccard Score output by both XGBOOST and single-layer neural network for each epoch during a training process. As shown in Figure S2, results obtained by these two methods are significantly correlated but there is still a slight difference in the number of epochs when Jaccard Score is at its maximum. Therefore, to train final D-CyPre with high Jaccard Score while speeding up model optimization, we use only faster method when tuning parameters and still train XGBOOST model at each epoch when building final model. In the case of CYP1A2, we plotted the loss curve when training the final model to confirm that the model was successfully trained.

Table S1. The descriptors calculated by the RDKit.

| Type | Descriptor Name | Description |
| --- | --- | --- |
| Atom Features | Atomic Number | The atomic number |
|  | Degree | The degree of the atom in the molecule including Hs |
|  | Formal Charge | The formal charge of the atom |
|  | Chirality | The chirality of the atom |
|  | Number Of Bonded Hydrogens | The total number of Hs (explicit and implicit) on the atom |
|  | Hybridization | The atom's hybridization |
|  | Aromaticity | The atom's aromaticity |
|  | Ring Membership | Whether or not the atom is in a ring of any size |
|  | Atomic Mass | Atomic mass |
| Bond Features | Bond Type (Single/Double/Rriple/Aromatic) | Bond type |
|  | Conjugation | Whether or not the bond is considered to be conjugated |
|  | Ring Membership | Whether or not the bond is in a ring of any size |
|  | Stereochemistry | The stereo configuration of the bond as a BondStereo |
| Molecular Features | MolWt | The average molecular weight of the molecule |
|  | NumHAcceptors | Number of Hydrogen Bond Acceptors |
|  | NumHDonors | Number of Hydrogen Bond Donors |
|  | MolLogP | Wildman-Crippen LogP value |
|  | TPSA | Topological polar surface area |
|  | LabuteASA | Returns Labute’s approximate surface area (ASA) for a molecule |

Table S2. Training results (Precision Mode) compared with CyProduct for nine CYP450 enzymes in EBoMD.

|  | 1A2 | 2A6 | 2B6 | 2C8 | 2C9 | 2C19 | 2D6 | 2E1 | 3A4 | WAvg^f^ |
| --- | --- | --- | --- | --- | --- | --- | --- | --- | --- | --- |
|  | Jaccard Score TP/(TP+FP+FN) | | | | | | | | | |
| D-CyPre^a^ | 0.534 | 0.751 | 0.625 | 0.784 | 0.680 | 0.617 | 0.545 | 0.728 | 0.622 | 0.630 |
| D-CyPre-val^b^ | 0.455 | 0.677 | 0.489 | 0.489 | 0.512 | 0.588 | 0.550 | 0.703 | 0.445 | 0.518 |
| CypBoM^c^ | 0.441 | 0.393 | 0.452 | 0.394 | 0.380 | 0.449 | 0.433 | 0.376 | 0.441 | 0.425 |
| D-CyPre-all^d^ | 0.560 | 0.747 | 0.652 | 0.760 | 0.692 | 0.612 | 0.560 | 0.744 | 0.615 | 0.636 |
| Random Predictor^e^ | 0.083 | 0.085 | 0.071 | 0.065 | 0.068 | 0.080 | 0.065 | 0.098 | 0.061 | 0.072 |
|  | Precision TP/(TP+FP) | | | | | | | | | |
| D-CyPre | 0.816 | 0.899 | 0.896 | 0.976 | 0.831 | 0.858 | 0.747 | 0.860 | 0.881 | 0.853 |
| D-CyPre-val | 0.855 | 0.894 | 0.830 | 0.745 | 0.758 | 0.796 | 0.843 | 0.867 | 0.766 | 0.808 |
| CypBoM | 0.659 | 0.602 | 0.663 | 0.800 | 0.520 | 0.638 | 0.576 | 0.514 | 0.624 | 0.620 |
| D-CyPre-all | 0.831 | 0.910 | 0.873 | 0.958 | 0.835 | 0.846 | 0.759 | 0.876 | 0.866 | 0.851 |
| Random Predictor | 0.090 | 0.093 | 0.076 | 0.069 | 0.073 | 0.085 | 0.069 | 0.108 | 0.065 | 0.078 |
|  | Recall TP/(TP+FN) | | | | | | | | | |
| D-CyPre | 0.607 | 0.820 | 0.674 | 0.799 | 0.790 | 0.688 | 0.668 | 0.825 | 0.679 | 0.705 |
| D-CyPre-val | 0.494 | 0.737 | 0.543 | 0.587 | 0.613 | 0.693 | 0.613 | 0.787 | 0.515 | 0.591 |
| CypBoM | 0.572 | 0.532 | 0.586 | 0.575 | 0.585 | 0.603 | 0.635 | 0.583 | 0.600 | 0.592 |
| D-CyPre-all | 0.632 | 0.807 | 0.720 | 0.786 | 0.802 | 0.689 | 0.682 | 0.832 | 0.680 | 0.714 |
| Random Predictor | 0.502 | 0.505 | 0.506 | 0.489 | 0.526 | 0.559 | 0.508 | 0.504 | 0.511 | 0.513 |
|  | F1 2$\times$Precision$\times$Recall/(Precision+Recall) | | | | | | | | | |
| D-CyPre | 0.696 | 0.858 | 0.769 | 0.879 | 0.810 | 0.764 | 0.705 | 0.842 | 0.767 | 0.770 |
| D-CyPre-val | 0.626 | 0.808 | 0.657 | 0.657 | 0.678 | 0.741 | 0.710 | 0.825 | 0.616 | 0.680 |
| CypBoM | 0.612 | 0.565 | 0.622 | 0.669 | 0.551 | 0.620 | 0.604 | 0.546 | 0.612 | 0.603 |
| D-CyPre-all | 0.718 | 0.855 | 0.789 | 0.864 | 0.818 | 0.759 | 0.718 | 0.853 | 0.762 | 0.775 |
| Random Predictor | 0.153 | 0.157 | 0.132 | 0.121 | 0.128 | 0.148 | 0.121 | 0.178 | 0.115 | 0.134 |

a: The results of D-CyPre on train set; b: The results of D-CyPre on validation set; c: The Cross-Validation Results of CypBoM on EBoMD; d: The results of D-CyPre on EBoMD; e: The results of the Random Predictor which identify SOMs with a 50% probability on EBoMD; f: The microaverage (weighted average, weighted by the number of SOMs) over the nine.

Table S3. Training results (Recall Mode) compared with CyProduct for nine CYP450 enzymes in EBoMD.

|  | 1A2 | 2A6 | 2B6 | 2C8 | 2C9 | 2C19 | 2D6 | 2E1 | 3A4 | WAvg^f^ |
| --- | --- | --- | --- | --- | --- | --- | --- | --- | --- | --- |
|  | Jaccard Score TP/(TP+FP+FN) | | | | | | | | | |
| D-CyPre^a^ | 0.606 | 0.771 | 0.644 | 0.788 | 0.688 | 0.650 | 0.575 | 0.729 | 0.646 | 0.657 |
| D-CyPre-val^b^ | 0.459 | 0.652 | 0.577 | 0.504 | 0.554 | 0.573 | 0.561 | 0.709 | 0.517 | 0.547 |
| CypBoM^c^ | 0.441 | 0.393 | 0.452 | 0.394 | 0.380 | 0.449 | 0.433 | 0.376 | 0.441 | 0.425 |
| D-CyPre-all^d^ | 0.597 | 0.759 | 0.644 | 0.774 | 0.685 | 0.660 | 0.588 | 0.742 | 0.656 | 0.660 |
| Random Predictor^e^ | 0.083 | 0.085 | 0.071 | 0.065 | 0.068 | 0.080 | 0.065 | 0.098 | 0.061 | 0.072 |
|  | Precision TP/(TP+FP) | | | | | | | | | |
| D-CyPre | 0.705 | 0.843 | 0.689 | 0.848 | 0.752 | 0.721 | 0.636 | 0.820 | 0.728 | 0.732 |
| D-CyPre-val | 0.584 | 0.827 | 0.652 | 0.702 | 0.678 | 0.662 | 0.664 | 0.830 | 0.657 | 0.673 |
| CypBoM | 0.659 | 0.602 | 0.663 | 0.800 | 0.520 | 0.638 | 0.576 | 0.514 | 0.624 | 0.620 |
| D-CyPre-all | 0.708 | 0.861 | 0.702 | 0.840 | 0.745 | 0.751 | 0.643 | 0.837 | 0.751 | 0.744 |
| Random Predictor | 0.090 | 0.093 | 0.076 | 0.069 | 0.073 | 0.085 | 0.069 | 0.108 | 0.065 | 0.078 |
|  | Recall TP/(TP+FN) | | | | | | | | | |
| D-CyPre | 0.810 | 0.900 | 0.907 | 0.918 | 0.890 | 0.869 | 0.857 | 0.868 | 0.852 | 0.864 |
| D-CyPre-val | 0.680 | 0.754 | 0.833 | 0.641 | 0.752 | 0.811 | 0.783 | 0.830 | 0.708 | 0.744 |
| CypBoM | 0.572 | 0.532 | 0.586 | 0.575 | 0.585 | 0.603 | 0.635 | 0.583 | 0.600 | 0.592 |
| D-CyPre-all | 0.792 | 0.865 | 0.887 | 0.907 | 0.896 | 0.844 | 0.872 | 0.868 | 0.838 | 0.854 |
| Random Predictor | 0.502 | 0.505 | 0.506 | 0.489 | 0.526 | 0.559 | 0.508 | 0.504 | 0.511 | 0.513 |
|  | F1 2$\times$Precision$\times$Recall/(Precision+Recall) | | | | | | | | | |
| D-CyPre | 0.754 | 0.871 | 0.783 | 0.882 | 0.815 | 0.788 | 0.730 | 0.843 | 0.785 | 0.792 |
| D-CyPre-val | 0.628 | 0.789 | 0.731 | 0.670 | 0.713 | 0.729 | 0.719 | 0.830 | 0.682 | 0.705 |
| CypBoM | 0.612 | 0.565 | 0.622 | 0.669 | 0.551 | 0.620 | 0.604 | 0.546 | 0.612 | 0.603 |
| D-CyPre-all | 0.748 | 0.863 | 0.784 | 0.872 | 0.814 | 0.795 | 0.740 | 0.852 | 0.792 | 0.794 |
| Random Predictor | 0.153 | 0.157 | 0.132 | 0.121 | 0.128 | 0.148 | 0.121 | 0.178 | 0.115 | 0.134 |

a: The results of D-CyPre on train set; b: The results of D-CyPre on validation set; c: The Cross-Validation Results of CyProduct on EBoMD; d: The results of D-CyPre on EBoMD; e: The results of the Random Predictor which identify SOMs with a 50% probability on EBoMD; f: The microaverage (weighted average, weighted by the number of SOMs) over the nine.Table S4. Results (Precision Mode) for nine CYP450 enzymes compared with Random Predictor on 68 reactants of EBoMD2.

|  | 1A2 | 2A6 | 2B6 | 2C8 | 2C9 | 2C19 | 2D6 | 2E1 | 3A4 | WAvg^c^ |
| --- | --- | --- | --- | --- | --- | --- | --- | --- | --- | --- |
|  | Jaccard Score TP/(TP+FP+FN) | | | | | | | | | |
| D-CyPre^a^ | 0.457 | 0.558 | 0.261 | 0.267 | 0.639 | 0.492 | 0.548 | 0.469 | 0.472 | 0.486 |
| Random Predictor^b^ | 0.049 | 0.067 | 0.047 | 0.047 | 0.051 | 0.040 | 0.055 | 0.136 | 0.055 | 0.054 |
|  | Precision TP/(TP+FP) | | | | | | | | | |
| D-CyPre | 0.685 | 0.906 | 0.444 | 0.593 | 0.852 | 0.750 | 0.735 | 0.958 | 0.643 | 0.717 |
| Random Predictor | 0.051 | 0.072 | 0.049 | 0.049 | 0.053 | 0.041 | 0.058 | 0.157 | 0.059 | 0.058 |
|  | Recall TP/(TP+FN) | | | | | | | | | |
| D-CyPre | 0.578 | 0.592 | 0.387 | 0.327 | 0.719 | 0.588 | 0.684 | 0.479 | 0.640 | 0.603 |
| Random Predictor | 0.500 | 0.500 | 0.500 | 0.500 | 0.500 | 0.500 | 0.500 | 0.500 | 0.500 | 0.500 |
|  | F1 2$\times$Precision$\times$Recall/(Precision+Recall) | | | | | | | | | |
| D-CyPre | 0.627 | 0.716 | 0.414 | 0.422 | 0.780 | 0.659 | 0.709 | 0.639 | 0.641 | 0.648 |
| Random Predictor | 0.093 | 0.126 | 0.090 | 0.090 | 0.097 | 0.077 | 0.103 | 0.239 | 0.105 | 0.102 |

a: The results of D-CyPre on EBoMD2; b: The results of the Random Predictor which identify SOMs with a 50% probability on EBoMD2; c: The microaverage (weighted average, weighted by the number of SOMs) over the nine.Table S5. Distribution of Non-SOMs/SOMs for nine CYP450 Isoforms in Data Sets.

|  | 1A2 | 2A6 | 2B6 | 2C8 | 2C9 | 2C19 | 2D6 | 2E1 | 3A4 |
| --- | --- | --- | --- | --- | --- | --- | --- | --- | --- |
|  | EBoMD2 | | | | | | | | |
| Reactants | 16 | 10 | 11 | 9 | 13 | 13 | 24 | 10 | 41 |
| SOMs | 64 | 49 | 31 | 49 | 64 | 51 | 158 | 48 | 236 |
| Non-SOMs | 1182 | 631 | 596 | 946 | 1134 | 1180 | 2581 | 258 | 3788 |
| Non-SOMs/SOMs | 18.47 | 12.88 | 19.23 | 19.31 | 17.72 | 23.14 | 16.34 | 5.38 | 16.05 |

Table S6. Results (Recall Mode) for nine CYP450 enzymes compared with Random Predictor on 68 reactants of EBoMD2.

|  | 1A2 | 2A6 | 2B6 | 2C8 | 2C9 | 2C19 | 2D6 | 2E1 | 3A4 | WAvg^c^ |
| --- | --- | --- | --- | --- | --- | --- | --- | --- | --- | --- |
|  | Jaccard Score TP/(TP+FP+FN) | | | | | | | | | |
| D-CyPre^a^ | 0.535 | 0.554 | 0.358 | 0.365 | 0.580 | 0.405 | 0.554 | 0.500 | 0.468 | 0.493 |
| Random Predictor^b^ | 0.049 | 0.067 | 0.047 | 0.047 | 0.051 | 0.040 | 0.055 | 0.136 | 0.055 | 0.054 |
|  | Precision TP/(TP+FP) | | | | | | | | | |
| D-CyPre | 0.593 | 0.816 | 0.463 | 0.519 | 0.734 | 0.507 | 0.615 | 1.000 | 0.570 | 0.627 |
| Random Predictor | 0.051 | 0.072 | 0.049 | 0.049 | 0.053 | 0.041 | 0.058 | 0.157 | 0.059 | 0.058 |
|  | Recall TP/(TP+FN) | | | | | | | | | |
| D-CyPre | 0.844 | 0.633 | 0.613 | 0.551 | 0.734 | 0.667 | 0.848 | 0.500 | 0.725 | 0.721 |
| Random Predictor | 0.500 | 0.500 | 0.500 | 0.500 | 0.500 | 0.500 | 0.500 | 0.500 | 0.500 | 0.500 |
|  | F1 2$\times$Precision$\times$Recall/(Precision+Recall) | | | | | | | | | |
| D-CyPre | 0.697 | 0.713 | 0.528 | 0.535 | 0.734 | 0.576 | 0.713 | 0.667 | 0.638 | 0.658 |
| Random Predictor | 0.093 | 0.126 | 0.090 | 0.090 | 0.097 | 0.077 | 0.103 | 0.239 | 0.105 | 0.102 |

a: The results of D-CyPre on EBoMD2; b: The results of the Random Predictor which identify SOMs with a 50% probability on EBoMD2; c: The microaverage (weighted average, weighted by the number of SOMs) over the nine.

Table S7. Training results of two kinds of molecular features.

|  | 1A2 | 2B6 |
| --- | --- | --- |
|  | Jaccard Score TP/(TP+FP+FN) | |
| D-CyPre-M^a^ | 0.709 | 0.805 |
| D-CyPre-val-M^b^ | 0.464 | 0.425 |
| D-CyPre-D^c^ | 0.845 | 0.855 |
| D-CyPre-val-D^d^ | 0.475 | 0.448 |
|  | Precision TP/(TP+FP) | |
| D-CyPre-M | 0.927 | 0.959 |
| D-CyPre-val-M | 0.758 | 0.767 |
| D-CyPre-D | 0.989 | 0.990 |
| D-CyPre-val-D | 0.832 | 0.885 |
|  | Recall TP/(TP+FN) | |
| D-CyPre-M | 0.751 | 0.834 |
| D-CyPre-val-M | 0.545 | 0.425 |
| D-CyPre-D | 0.854 | 0.862 |
| D-CyPre-val-D | 0.526 | 0.475 |
|  | F1 2$\times$Precision$\times$Recall/(Precision+Recall) | |
| D-CyPre-M | 0.830 | 0.892 |
| D-CyPre-val-M | 0.634 | 0.547 |
| D-CyPre-D | 0.917 | 0.922 |
| D-CyPre-val-D | 0.645 | 0.618 |

a: The results of D-CyPre (including molecular features generated by a fixed rule) on train set; b: The results of D-CyPre (including molecular features generated by a fixed rule) on validation set; c: The results of D-CyPre (including molecular features calculated by D-MPNN) on train set; d: The results of D-CyPre (including molecular features calculated by D-MPNN) on validation set.

Table S8. Training results (Precision Mode) of D-CyPre (include molecular features) for nine CYP450 enzymes in EBoMD and EBoMD2.

|  | 1A2 | 2A6 | 2B6 | 2C8 | 2C9 | 2C19 | 2D6 | 2E1 | 3A4 | WAvg^f^ |
| --- | --- | --- | --- | --- | --- | --- | --- | --- | --- | --- |
|  | Jaccard Score TP/(TP+FP+FN) | | | | | | | | | |
| D-CyPre^a^ | 0.845 | 0.919 | 0.855 | 0.832 | 0.882 | 0.818 | 0.595 | 0.870 | 0.791 | 0.804 |
| D-CyPre-val^b^ | 0.475 | 0.695 | 0.448 | 0.500 | 0.527 | 0.611 | 0.536 | 0.704 | 0.469 | 0.528 |
| D-CyPre-all^c^ | 0.826 | 0.880 | (-)^e^ | 0.760 | 0.870 | 0.827 | (-) | 0.822 | 0.765 | (-) |
| D-CyPre-test^d^ | 0.593 | 0.549 | (-) | 0.281 | 0.600 | 0.323 | (-) | 0.377 | 0.462 | (-) |
|  | Precision TP/(TP+FP) | | | | | | | | | |
| D-CyPre | 0.989 | 0.994 | 0.990 | 0.990 | 0.978 | 0.966 | 0.770 | 0.963 | 0.968 | 0.949 |
| D-CyPre-val | 0.832 | 0.953 | 0.885 | 0.729 | 0.751 | 0.823 | 0.843 | 0.844 | 0.769 | 0.811 |
| D-CyPre-all | 0.978 | 0.998 | (-) | 0.983 | 0.974 | 0.968 | (-) | 0.950 | 0.962 | (-) |
| D-CyPre-test | 0.699 | 0.933 | (-) | 0.667 | 0.804 | 0.600 | (-) | 0.639 | 0.676 | (-) |
|  | Recall TP/(TP+FN) | | | | | | | | | |
| D-CyPre | 0.854 | 0.924 | 0.862 | 0.839 | 0.900 | 0.842 | 0.723 | 0.900 | 0.812 | 0.836 |
| D-CyPre-val | 0.526 | 0.719 | 0.475 | 0.614 | 0.639 | 0.704 | 0.596 | 0.809 | 0.546 | 0.604 |
| D-CyPre-all | 0.841 | 0.881 | (-) | 0.770 | 0.891 | 0.850 | (-) | 0.859 | 0.788 | (-) |
| D-CyPre-test | 0.797 | 0.571 | (-) | 0.327 | 0.703 | 0.412 | (-) | 0.479 | 0.593 | (-) |
|  | F1 2$\times$Precision$\times$Recall/(Precision+Recall) | | | | | | | | | |
| D-CyPre | 0.917 | 0.958 | 0.922 | 0.908 | 0.937 | 0.900 | 0.746 | 0.930 | 0.883 | 0.889 |
| D-CyPre-val | 0.645 | 0.820 | 0.618 | 0.667 | 0.690 | 0.759 | 0.698 | 0.826 | 0.639 | 0.689 |
| D-CyPre-all | 0.904 | 0.936 | (-) | 0.864 | 0.931 | 0.905 | (-) | 0.902 | 0.866 | (-) |
| D-CyPre-test | 0.745 | 0.708 | (-) | 0.439 | 0.750 | 0.489 | (-) | 0.548 | 0.632 | (-) |

a: The results of D-CyPre on train set; b: The results of D-CyPre on validation set; c: The results of D-CyPre on EBoMD; d: The results of D-CyPre on EBoMD2; e: Since the new model did not perform better in validation set, test set was not calculated; f: The microaverage (weighted average, weighted by the number of SOMs) over the nine.Table S9. Training results (Recall Mode) of D-CyPre (include molecular features) for nine CYP450 enzymes in EBoMD and EBoMD2.

|  | 1A2 | 2A6 | 2B6 | 2C8 | 2C9 | 2C19 | 2D6 | 2E1 | 3A4 | WAvg^f^ |
| --- | --- | --- | --- | --- | --- | --- | --- | --- | --- | --- |
|  | Jaccard Score TP/(TP+FP+FN) | | | | | | | | | |
| D-CyPre^a^ | 0.872 | 0.915 | 0.725 | 0.811 | 0.842 | 0.835 | 0.614 | 0.847 | 0.776 | 0.793 |
| D-CyPre-val^b^ | 0.501 | 0.708 | 0.550 | 0.506 | 0.545 | 0.619 | 0.559 | 0.689 | 0.506 | 0.554 |
| D-CyPre-all^c^ | 0.842 | 0.907 | (-)^e^ | 0.816 | (-) | 0.829 | (-) | (-) | (-) | (-) |
| D-CyPre-test^d^ | 0.571 | 0.636 | (-) | 0.328 | (-) | 0.463 | (-) | (-) | (-) | (-) |
|  | Precision TP/(TP+FP) | | | | | | | | | |
| D-CyPre | 0.970 | 0.965 | 0.740 | 0.911 | 0.888 | 0.923 | 0.711 | 0.909 | 0.831 | 0.863 |
| D-CyPre-val | 0.798 | 0.934 | 0.633 | 0.700 | 0.687 | 0.748 | 0.724 | 0.847 | 0.616 | 0.717 |
| D-CyPre-all | 0.957 | 0.956 | (-) | 0.922 | (-) | 0.920 | (-) | (-) | (-) | (-) |
| D-CyPre-test | 0.658 | 0.854 | (-) | 0.679 | (-) | 0.660 | (-) | (-) | (-) | (-) |
|  | Recall TP/(TP+FN) | | | | | | | | | |
| D-CyPre | 0.897 | 0.946 | 0.973 | 0.881 | 0.942 | 0.897 | 0.818 | 0.925 | 0.921 | 0.905 |
| D-CyPre-val | 0.574 | 0.746 | 0.809 | 0.647 | 0.725 | 0.781 | 0.710 | 0.787 | 0.738 | 0.715 |
| D-CyPre-all | 0.875 | 0.946 | (-) | 0.876 | (-) | 0.894 | (-) | (-) | (-) | (-) |
| D-CyPre-test | 0.813 | 0.714 | (-) | 0.388 | (-) | 0.608 | (-) | (-) | (-) | (-) |
|  | F1 2$\times$Precision$\times$Recall/(Precision+Recall) | | | | | | | | | |
| D-CyPre | 0.932 | 0.955 | 0.841 | 0.896 | 0.914 | 0.910 | 0.761 | 0.917 | 0.874 | 0.882 |
| D-CyPre-val | 0.668 | 0.829 | 0.710 | 0.672 | 0.705 | 0.764 | 0.717 | 0.816 | 0.672 | 0.711 |
| D-CyPre-all | 0.914 | 0.951 | (-) | 0.898 | (-) | 0.907 | (-) | (-) | (-) | (-) |
| D-CyPre-test | 0.727 | 0.778 | (-) | 0.494 | (-) | 0.633 | (-) | (-) | (-) | (-) |

a: The results of D-CyPre on train set; b: The results of D-CyPre on validation set; c: The results of D-CyPre on EBoMD; d: The results of D-CyPre on EBoMD2; e: Since the new model did not perform better in validation set, test set was not calculated; f: The microaverage (weighted average, weighted by the number of SOMs) over the nine.Table S10. Parameters of XGBOOST on nine CYP450 enzymes.

| Enzymes | $\mathrm{loss}_{p}$^e^ | $\mathrm{loss}_{n}$^f^ | Max Depth | Colsample Bytree | Reg Lambda | N Estimators | Scale Pos Weight |
| --- | --- | --- | --- | --- | --- | --- | --- |
| 1A2-P^a^ | 1 | 1 | 9 | 0.85 | 0 | 1000 | 1.000 |
| 2A6-P^a^ | 1.5 | 1 | 7 | 0.55 | 0 | 1000 | 1.000 |
| 2B6-P^a^ | 1.5 | 1 | 9 | 0.90 | 1 | 1000 | 1.000 |
| 2C8-P^a^ | 1.5 | 1 | 7 | 0.55 | 0 | 1000 | 1.000 |
| 2C9-P^a^ | 1 | 1 | 9 | 0.75 | 0 | 1000 | 1.000 |
| 2C19-P^a^ | 1 | 1 | 8 | 0.80 | 0 | 1000 | 1.000 |
| 2D6-P-O^b^ | 1 | 1 | 5 | 0.80 | 0 | 700 | 1.000 |
| 2E1-P^a^ | 1.5 | 1 | 8 | 0.85 | 1 | 1000 | 1.000 |
| 3A4-P^a^ | 1 | 1.5 | 10 | 0.75 | 0 | 1000 | 1.000 |
| 1A2-R^c^ | 1 | 1 | 9 | 0.85 | 0 | 1000 | 0.663 |
| 2A6-R^c^ | 1.5 | 1 | 7 | 0.55 | 0 | 1000 | 0.529 |
| 2B6-R^c^ | 1 | 1 | 9 | 0.90 | 1 | 1000 | 0.171 |
| 2C8-R^c^ | 1.5 | 1 | 7 | 0.55 | 0 | 1000 | 0.500 |
| 2C9-R^c^ | 1.5 | 1 | 9 | 0.75 | 0 | 1000 | 0.490 |
| 2C19-R^c^ | 1 | 1 | 8 | 0.80 | 0 | 1000 | 0.551 |
| 2D6-R-O^d^ | 1 | 1 | 5 | 0.80 | 0 | 700 | 0.274 |
| 2E1-R-O^d^ | 1 | 1.5 | 8 | 0.70 | 1 | 700 | 0.715 |
| 3A4-R-O^d^ | 1 | 1 | 9 | 0.80 | 0 | 1000 | 0.473 |

Min Child Weight is set to 2 for all models. a: Parameters of nine enzymes in Precision Mode (including molecular features calculated by D-MPNN); b: Parameters of nine enzymes in Precision Mode; c: Parameters of nine enzymes in Recall Mode (including molecular features calculated by D-MPNN); d: Parameters of nine enzymes in Precision Mode; e: The weight of $\mathrm{loss}_{p}$; f: The weight of $\mathrm{loss}_{n}$.Table S11. Training results of two methods for the 1A2 in EBoMD.

|  | 1A2 |
| --- | --- |
|  | Jaccard Score TP/(TP+FP+FN) |
| D-CyPre^a^ | 0.534 |
| D-CyPre-one^b^ | 0.609 |
| D-CyPre-val^c^ | 0.455 |
| D-CyPre-one-val^d^ | 0.317 |
|  | Precision TP/(TP+FP) |
| D-CyPre | 0.816 |
| D-CyPre-one | 0.845 |
| D-CyPre-val | 0.855 |
| D-CyPre-one-val | 0.665 |
|  | Recall TP/(TP+FN) |
| D-CyPre | 0.607 |
| D-CyPre-one | 0.686 |
| D-CyPre-val | 0.494 |
| D-CyPre-one-val | 0.377 |
|  | F1 2$\times$Precision$\times$Recall/(Precision+Recall) |
| D-CyPre | 0.696 |
| D-CyPre-one | 0.757 |
| D-CyPre-val | 0.626 |
| D-CyPre-one-val | 0.481 |

a: The results of D-CyPre on train set; b: The results of D-CyPre on train set (only 1A2); c: The results of D-CyPre on validation set; d: The results of D-CyPre on validation set (only 1A2).Table S12. Training results of two methods for the 2B6 and 2C9 in EBoMD.

|  | 2B6 | 2C9 |
| --- | --- | --- |
|  | Jaccard Score TP/(TP+FP+FN) | |
| D-CyPre-val^a^ | 0.489 | 0.512 |
| D-CyPre-all^b^ | 0.497 | 0.524 |
|  | Precision TP/(TP+FP) | |
| D-CyPre-val | 0.830 | 0.758 |
| D-CyPre-all | 0.756 | 0.767 |
|  | Recall TP/(TP+FN) | |
| D-CyPre-val | 0.543 | 0.613 |
| D-CyPre-all | 0.592 | 0.623 |
|  | F1 2$\times$Precision$\times$Recall/(Precision+Recall) | |
| D-CyPre-val | 0.657 | 0.678 |
| D-CyPre-all | 0.664 | 0.688 |

a: The results of D-CyPre on validation set; b: The results of D-CyPre (Training the models of the atoms and bonds separately) on validation set.Table S13. Testing results of the D-CyPre (include non-reactants) for nine CYP450 enzymes in EBoMD2.

|  | 1A2 | 2A6 | 2B6 | 2C8 | 2C9 | 2C19 | 2D6 | 2E1 | 3A4 | WAvg^c^ |
| --- | --- | --- | --- | --- | --- | --- | --- | --- | --- | --- |
|  | Jaccard Score TP/(TP+FP+FN) | | | | | | | | | |
| D-CyPre-P^a^ | 0.210 | 0.424 | 0.129 | 0.168 | 0.357 | 0.156 | 0.478 | 0.274 | 0.300 | 0.316 |
| D-CyPre-R^b^ | 0.210 | 0.422 | 0.098 | 0.184 | 0.281 | 0.217 | 0.351 | 0.282 | 0.363 | 0.307 |
|  | Precision TP/(TP+FP) | | | | | | | | | |
| D-CyPre-P | 0.222 | 0.622 | 0.162 | 0.258 | 0.414 | 0.200 | 0.614 | 0.390 | 0.378 | 0.405 |
| D-CyPre-R | 0.220 | 0.507 | 0.104 | 0.216 | 0.313 | 0.252 | 0.374 | 0.393 | 0.421 | 0.351 |
|  | Recall TP/(TP+FN) | | | | | | | | | |
| D-CyPre-P | 0.797 | 0.571 | 0.387 | 0.327 | 0.719 | 0.412 | 0.684 | 0.479 | 0.593 | 0.593 |
| D-CyPre-R | 0.813 | 0.714 | 0.613 | 0.551 | 0.734 | 0.608 | 0.848 | 0.500 | 0.725 | 0.720 |
|  | F1 2$\times$Precision$\times$Recall/(Precision+Recall) | | | | | | | | | |
| D-CyPre-P | 0.347 | 0.595 | 0.228 | 0.288 | 0.525 | 0.269 | 0.647 | 0.430 | 0.462 | 0.469 |
| D-CyPre-R | 0.346 | 0.593 | 0.178 | 0.310 | 0.439 | 0.356 | 0.519 | 0.440 | 0.533 | 0.463 |

a: The results of D-CyPre (Precision Mode) on test set; b: The results of D-CyPre (Recall Mode) on test set; c: The microaverage (weighted average, weighted by the number of SOMs) over the nine.

Table S14. The running time of each model in predicting metabolites under nine CYP450 subtypes of a single compound.

|  | D-CyPre | CyProduct | Biotransformer |
| --- | --- | --- | --- |
| Time (s) | 6.75 | 18.04 | 16.15 |


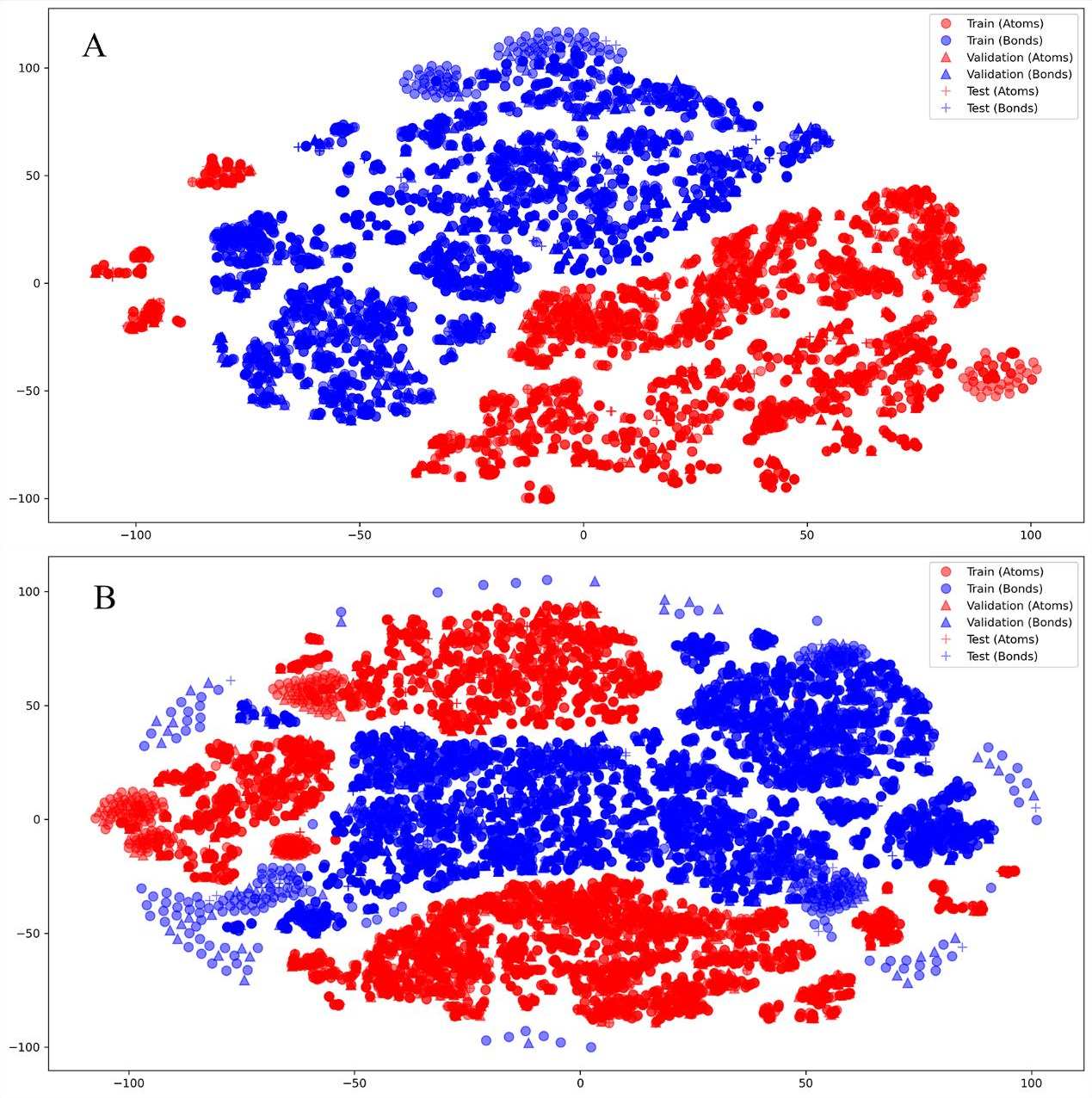


Figure S1. Visualization (by t-SNE) of features of the atoms and bonds of the 2B6 (A) and 2C9 (B).


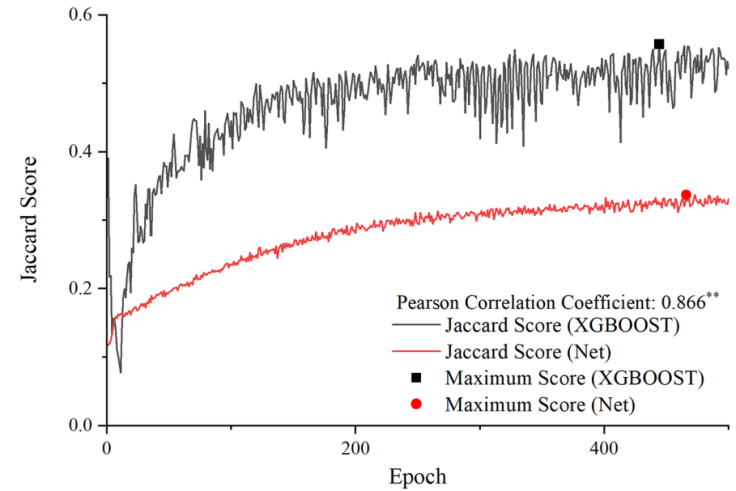


Figure S2. Training results of the two methods (shown for a specific instance of CYP2A6).


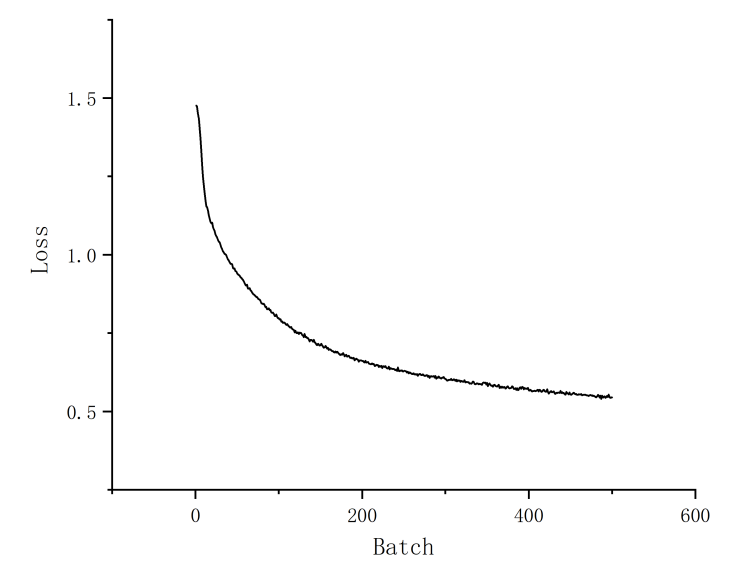


Figure S3. The loss curves of the model (shown for a specific instance of CYP1A2).

Software

Step. 1 import your data.


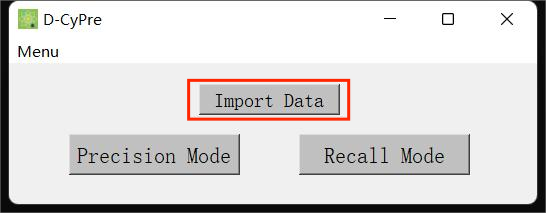


Figure S3. Import your data.

Your data should be a CSV table (such as the csv that under the sample folder)


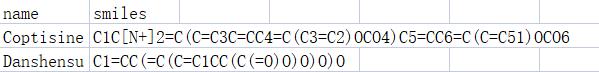


Figure S4. Example data.

Step. 2 Selecting an Analysis Type.


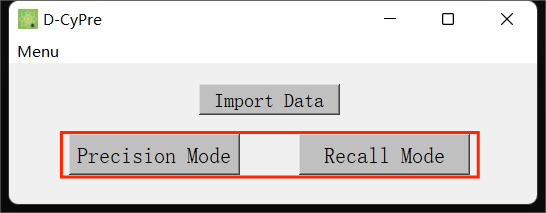


Figure S5. Selecting an Analysis Type.

Step. 3 Get the results of your analysis.


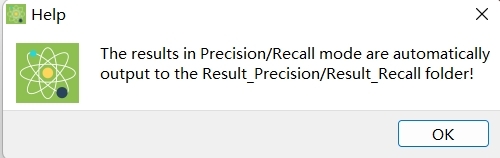


Figure S6. Get the results of your analysis.

Graphical abstract


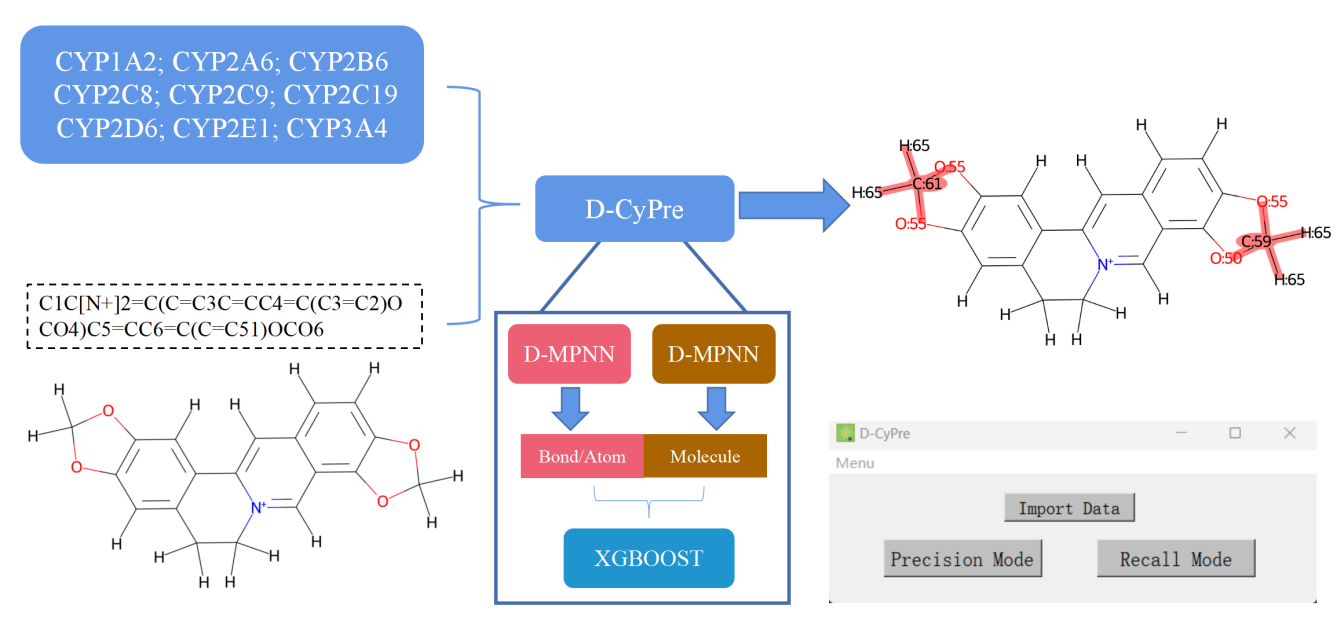

Supplement: Supplemental Information 1 — The charts referenced in the article and a comparative analysis of the performance of feature generators trained on data from either a single enzyme or a set of nine enzymes. This also investigates the effect of training models using the features of atoms and bonds separately. [file peerj-cs-10-2040-s001.docx]
