# Supplementary figures and images for: D-CyPre: a machine learning-based tool for accurate prediction of human CYP450 enzyme metabolic sites
Source: PeerJ Comput Sci. 2024 May 7;10:e2040. doi: 10.7717/peerj-cs.2040 (PMC11157575; doi:10.7717/peerj-cs.2040)

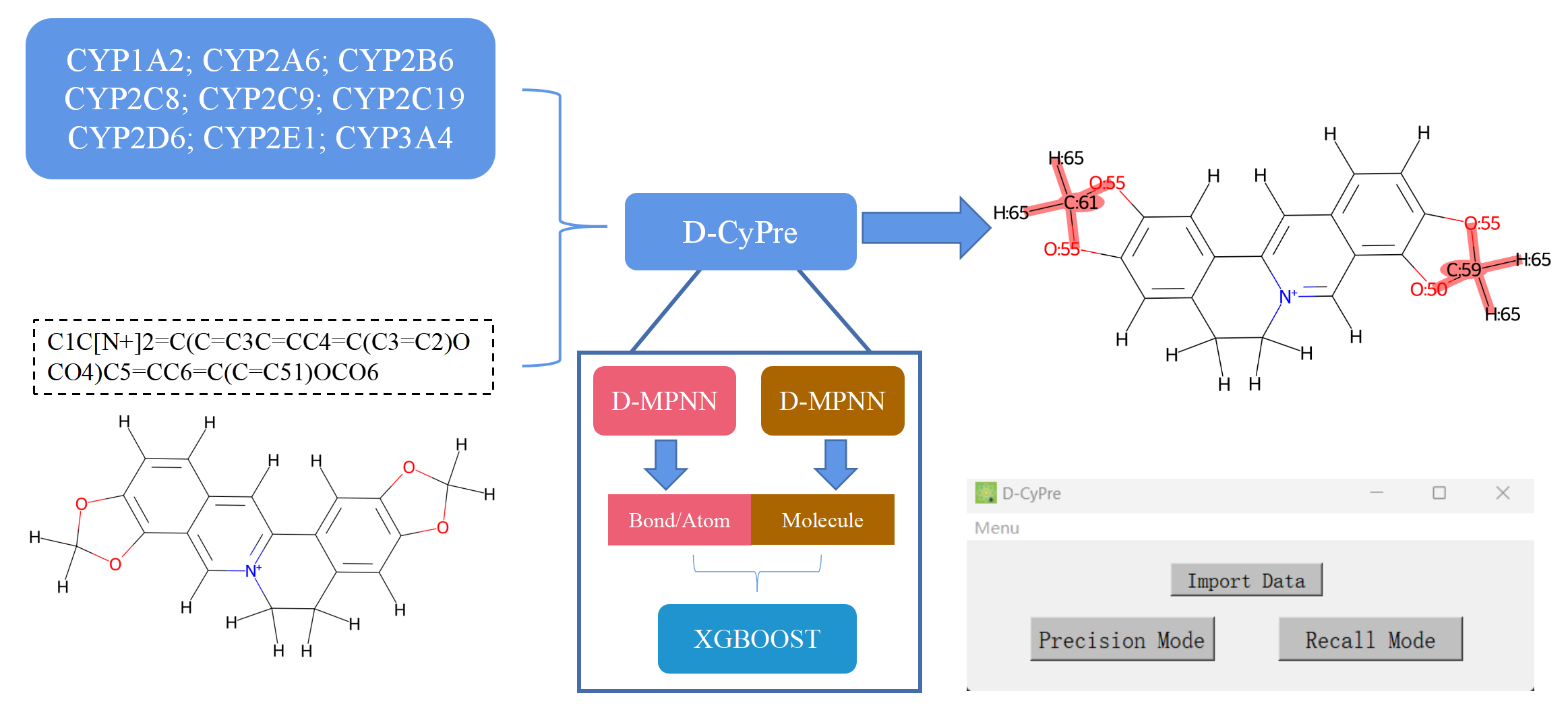

Supplement: Supplemental Information 2 — The D-CyPre software is freely accessible at https://github.com/67520/D-CyPre. The data set (EBoMD.sdf and EBoMD2.sdf) comes from CyProduct: A Software Tool for Accurately Predicting the Byproducts of Human Cytochrome P450 Metabolism. doi: https://doi.org/10.1021/acs.jcim.1c00144. [file peerj-cs-10-2040-s002.zip › supplementary2/overview.png]
